# Emergency team calls for critically ill non-trauma patients in the emergency department: an observational study

**DOI:** 10.1186/s13049-015-0159-2

**Published:** 2015-10-06

**Authors:** Søren Marker Jensen, Hien Quoc Do, Søren W. Rasmussen, Lars S. Rasmussen, Thomas Andersen Schmidt

**Affiliations:** Emergency Department, Holbaek Hospital, Copenhagen University Hospital, ᅟ, Denmark; Department of Anaesthesia, Centre of Head and Orthopaedics, Rigshospitalet, Copenhagen University Hospital, Copenhagen, Denmark

**Keywords:** Emergency department, Critically ill, Clinical assessment, Diagnoses, Death/mortality, Intensive care

## Abstract

**Background:**

Handling critically ill patients is a complex task for Emergency Department (ED) personnel. Initial treatment is of major importance and requires adequately experienced ED doctors to initiate and decide for the right medical or surgical treatment. Our aim was, with regard to clinical presentation, management and mortality to describe adult non-trauma patients that upon ED arrival elicited emergency team calls.

**Methods:**

An observational study of adult patients (≥18 years) admitted to a regional ED with conditions that elicited acute team activation and additional emergency team consultation calls for non-ED specialist physicians. Emergency team calls were two-tiered with ‘orange’ and ‘red’ calls. Additionally, intensive care unit (ICU) admission charts were reviewed to identify the total number of adult non-trauma and non-cardiac arrest patients admitted to the ICU from the ED during the study period.

**Results:**

A total of 109 emergency team calls were triggered (79 orange and 30 red), comprising 66 (60.6 %) men and 43 women, with a median age of 64 years. Patients presented with: 4 Airway, 27 Breathing, 41 Circulation, 31 Disability, 2 Exposure and 4 Other problems. Overall, 58/109 (53.2 %) patients were admitted to the ICU, while 20/109 (18.3 %) patients were deemed ineligible for ICU admission. 30-day mortality was 34/109 (31.2 %), and circulatory problems were the most frequent cause of death (61.8 %, *p* = 0.02). Patients who died were significantly older than those who survived (*p* = 0.004). Additionally, 115 adult patients were admitted to the ICU directly from the ED without eliciting an emergency team call during the study period. These patients mainly comprised patients who were intoxicated, were unconscious or had respiratory failure.

**Conclusion:**

The majority of emergency team call patients presented with circulatory, disability and breathing problems. Half of the patients were admitted to the ICU, although a high rate of patients was deemed ineligible for ICU admission. 30-day mortality was considerable and circulatory related illnesses were associated with increased short-term mortality.

**Electronic supplementary material:**

The online version of this article (doi:10.1186/s13049-015-0159-2) contains supplementary material, which is available to authorized users.

## Background

Handling critically ill patients is a complex task for ED personnel. Initial treatment is of major importance and requires adequately experienced ED doctors to initiate the appropriate medical or surgical treatment. Many EDs in Western countries are primarily manned by younger and less experienced physicians [1, 2], and many EDs do not have or use well-defined admission criteria and triage systems for acute critically ill medical patients [3]. At our ED, initial assessment of critically ill patients is always performed with the attendance of a senior specialist ED physician. As advocated internationally, physicians in our department hold the possibility of activating an alert system eliciting a so-called emergency team call in severe cases, to facilitate further personnel resources from other hospital departments, e.g. anaesthesiologists [3–5].

At our ED there are two types of emergency team calls termed ‘red’ or ‘orange’ calls, respectively. Orange emergency team calls are triggered by the coordinating senior ED physician if initial treatment in the ED does not achieve successful stabilisation of a patient’s condition. Accordingly, a consultant anaesthesiologist and the attending medical or surgical physician, depending on the condition at hand, will join the ED acute team. Red emergency team calls are elicited prior to ambulance arrival by the coordinating senior ED physician in anticipation of the need for advanced airway management following report by telephone of the on-site paramedic. In case of a red emergency team call, a consultant anaesthesiologist, a nurse anaesthetist and an attending physician from the department of internal medicine will support the ED acute team.

In general, ED patients rarely need intensive care. But some critically ill patients may benefit from intensive care unit (ICU) admittance. ICU resources are expensive and therefore limited, and for optimal hospital resource utilisation, ICU admittance should accordingly be reserved for appropriate patients [6–12]. While there is considerable literature about ED patients in general, only few studies have described the most critically ill ED patients. We regard the emergency team call patients as some of the most critically ill patients in the ED setting, that require immediate attention and treatment in order to survive.

With regard to clinical presentation, management and mortality our aim was to describe adult non-trauma patients that upon ED arrival elicited emergency team calls.

## Methods

This study was conducted at the ED of a secondary emergency hospital in the Western part of Zealand, Denmark. Approximately 50 non-trauma patients are admitted to the ED daily.

### Study population

Data were collected retrospectively in a 1.5-year period between 14 April 2012 and 14 October 2013. We included all critically ill adult medical and surgical non-trauma patients (age ≥ 18 years) admitted acutely to our regional ED who triggered an ED acute team and emergency team call activation. Additionally, ICU admission charts were reviewed to identify the total number of adult non-trauma and non-cardiac arrest patients admitted to the ICU from the ED during the study period.

Patients with prehospital signs of ST segment elevation myocardial infarction (STEMI) on electrocardiography (ECG) were taken directly to a percutaneous coronary intervention (PCI) facility in Copenhagen, Denmark. Resuscitation teams for in-hospital and out-of-hospital cases of cardiac arrest were per protocol activated independently of the above emergency team calls, and dedicated cardiac arrest calls were thus not included in this study. However, three patients had cardiac arrest in the ED immediately upon hospital arrival, when the emergency team call team was already elicited/assembled. These three patients were therefore included in the study.

### Data collection

Data were collected from the in-hospital electronic patient charts with regard to: patient demographics, diagnosis codes, medical/surgical procedures, route of admission, length of stay (total and ICU), 24-h mortality and 30-day mortality. Furthermore, the diagnosis codes were stratified according to the Airway, Breathing, Circulation, Disability, Exposure (ABCDE) acuity approach (by SMJ, HQD and TAS) according to the patients’ predominant clinical problem in order to elucidate possible illness patterns. A category named ‘Other’ was included for conditions that were unsuitable for Airway, Breathing, Circulation, Disability, Exposure (ABCDE) classification.

### Statistics

Continuous data were reported as median with interquartile range (IQR) and compared using the Wilcoxon rank-sum test. Categorical data were reported as number with percentage (%) and compared using Fishers exact test or *χ*^2^ test as appropriate.

Data analysis was performed using SPSS Statistics 22 (IBM Corp., Somers, NY, USA). *p* < 0.05 was considered statistically significant.

### Ethics

According to Danish law, informed consent and approval from the ethics committee were not required for this study. Permissions from the Danish Data Protection Agency (12–000179) and the National Board of Health (3–3013–600/1) were obtained prior to the study.

## Results

A total of 109 emergency team calls were triggered (79 orange and 30 red) during the study period (Tables 1 and 2), comprising 66 (60.6 %) men and 43 women, with a median age of 64 (IQR: 50–79) years. The most common problems were circulatory (37.5 %) and disability related (28.4 %). Around a quarter of the study population had breathing related diagnoses (24.8 %). Both airway related problems and diagnosis categories classified as Other amounted to 3.7 %. Exposure problems amounted to 1.8 %.

**Table 1:** Characteristics of 109 critically ill adult non-trauma patients with regard to 30-day mortality

		Patients who survived 30 days from admission (*n* = 75)	Patients who did not survive 30 days from admission (*n* = 34)	*p*
Patient demographics				
	Age (years)	61 (46–73)	71 (62–80)	0.004
	Male gender	43 (57.3)	23 (67.6)	0.31
Diagnosis category				0.02
	Airway	4 (5.3)	0 (0.0)	
	Breathing	20 (26.7)	7 (20.6)	
	Circulation	20 (26.7)	21 (61.8)	
	Disability	25 (33.3)	6 (17.6)	
	Exposure	2 (2.7)	0 (0.0)	
	Other	4 (5.3)	0 (0.0)	
Route of admission				0.62
	112 call	64 (85.3)	27 (79.4)	
	On-call doctor service	7 (9.3)	3 (8.8)	
	General practitioner	1 (1.3)	2 (5.9)	
	Self-admission	2 (2.7)	1 (2.9)	
	Outpatient clinic	1 (1.3)	1 (2.9)	
Emergency team call type				0.22
	Red	18 (24.0)	12 (35.3)	
	Orange	57 (76.0)	22 (64.7)	
Intensive care unit				
	Considered ineligible	2 (2.7)	18 (52.9)	<0.0001
	Admission	43 (57.3)	15 (44.1)	0.20
Length of stay				
	Total	5 (2–10)	2.5 (1–9.3)	0.14
	ICU	1 (0–2)	0 (0–2)	0.56

**Table 2:** Distribution of Airway, Breathing, Circulation, Disability, Exposure (ABCDE)-categories and medical/surgical diagnoses among 109 critically ill adult non-trauma patients

Category	Diagnosis	Number	Percent
Airway		4	3.7
	Anaphylaxis	2	50.0
	FBAO	1	25.0
	Hereditary angiooedema	1	25.0
Breathing		27	24.8
	Pneumonia	13	48.1
	COLD	6	22.2
	Pulmonary oedema	6	22.2
	Pulmonary embolism	2	7.4
Circulation		41	37.6
	Haemorrhage	11	26.8
	MI	9	22.0
	Sepsis (non-respiratory)	6	14.6
	Arrhythmia	4	9.8
	Acute kidney failure	3	7.3
	Cardiac arrest	3	7.3
	Dehydration	2	4.9
	Ketoacidosis	2	4.9
	Hypotension	1	2.4
Disability		31	28.4
	Intoxication	10	32.3
	Seizures	8	25.8
	Cerebral haemorrhage	7	22.6
	Metabolic	2	6.5
	Stroke	2	6.5
	Hypoglycemia	1	3.2
	Psychiatric problems	1	3.2
Exposure		2	1.8
	Hypothermia	1	50.0
	Smoke inhalation	1	50.0
Other		4	3.7
	Pain	3	75.0
	Abscess	1	25.0

*FBAO* foreign body airway obstruction, *COLD* Chronic obstructive lung disease, *MI* Myocardial infarction

Within 24 h of hospital admission, tracheal intubation was performed in 24 cases (22.0 %). Emergency ultrasound was performed on 15 patients (13.8 %), consisting of 12 echocardiographies and 3 ultrasound examinations of the abdomen. A total of 35 patients (32.1 %) had a Computed Tomography (CT) scan performed, including 20 cerebral CTs and 15 CTs of the thorax/abdomen. Furthermore, 9 patients (8.3 %) needed surgical procedures (PCI treatment not included).

A total of 20/109 (18.3 %) patients were deemed ineligible for ICU admission, and 58/109 (53.2 %) patients were admitted to the ICU. Median total length of stay (LOS) in hospital was 5 (IQR: 2–10) days, while median ICU LOS was 1 (IQR: 0–2) day. Mechanical ventilation in the ICU was applied in a total of 25 patients (22.9 %), with a median duration of 1 day.

Transfer of patients to other health care facilities was performed in 16 cases (14.7 %). These included 14 patient transfers to more specialised treatment options elsewhere, and two transfers to rehabilitation facilities. The most frequent causes for patient transfer were need of PCI (4 cases) and neurosurgical treatment (3 cases).

Twenty-four-hour mortality was 12/109 (11.0 %). Deaths due to circulatory problems comprised 8/12 (66.7 %), however, no statistically significant difference in 24 h mortality was found between the Airway, Breathing, Circulation, Disability, Exposure (ABCDE) categories (*p* = 0.54).

**Fig. 1:**
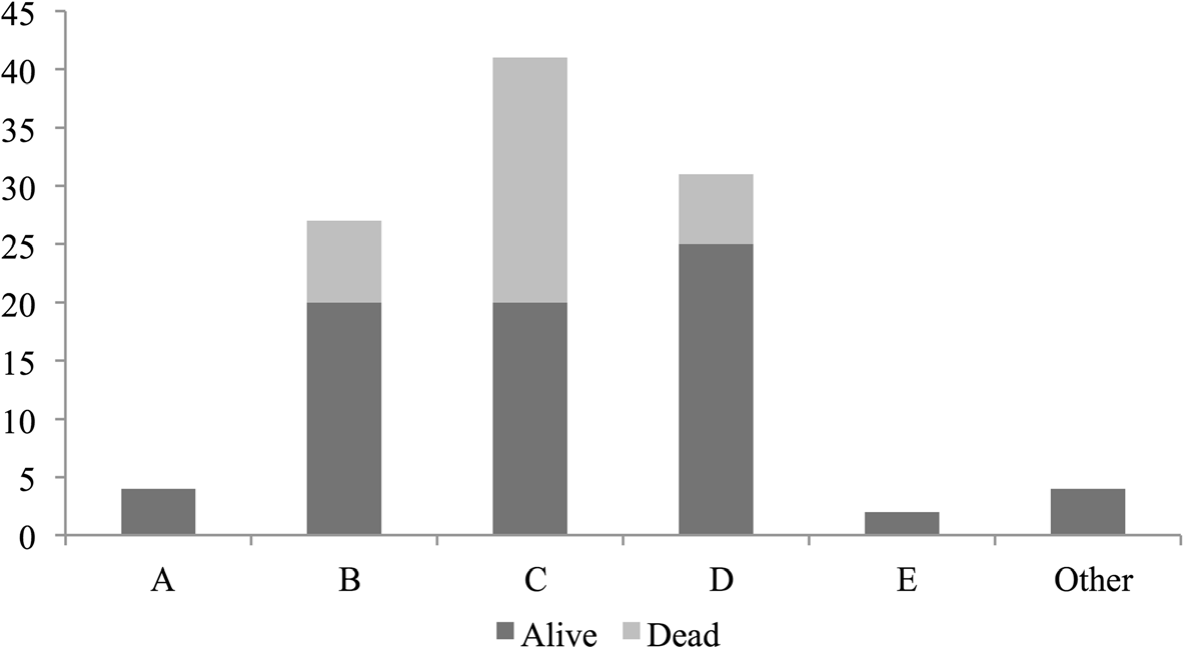
Distribution, in absolute numbers, of patient diagnoses according to Airway (**a**), Breathing (**b**), Circulation (**c**), Disability (**d**), Exposure (**e**) and Other categories and the associated 30-day mortality

Thirty-day mortality was 34/109 (31.2 %), with significant differences according to Airway, Breathing, Circulation, Disability, Exposure (ABCDE) presentations (*p* = 0.02) (Table 1). Circulatory problems were most frequently associated with death (61.8 %) (Fig. 1) (see Additional file 1: Table S3). This link remains statistically significant even if the three deaths categorised as cardiac arrest were to be omitted (*p* = 0.04). Patients who died within 30-days of admission were significantly older than those who survived (71 vs. 61 years respectively, *p* = 0.004). Emergency team call type (*p* = 0.22), gender (*p* = 0.31) and ICU admission (*p* = 0.20) were not significant risk factors of 30-day mortality. Among patients dying within 30 days following admission, 52.9 % were considered ineligible for intensive care treatment, at the initial ED assessment by the assembled team of physicians, due to high age, severe comorbidities etc.

Additionally, a total of 115 adult patients were admitted to the ICU directly from the ED without eliciting an orange or red emergency team call during the study period. These patients mainly comprised patients who suffered from intoxication or were unconscious including postictal status (disability problems: 43.5 %), and patients with respiratory problems, primarily exacerbation of chronic obstructive lung disease (COPD) (breathing problems: 36.5 %).

## Discussion

In this study we sought to describe adult non-trauma patients eliciting emergency team calls in a secondary hospital ED. On average one emergency team call was elicited per week. The majority of these patients presented with circulatory, disability and breathing problems, respectively. Half of these patients were admitted to the ICU. A total of 20/109 patients (18.3 %) were deemed ineligible for ICU admission. The substantial proportion of patients being deemed ineligible for ICU treatment does not necessarily indicate critical illness, but only that a discussion about ICU suitability had taken place. Thirty-day mortality among all patients eliciting emergency team call was considerable (31.2 %), and increasing age and circulatory related illnesses were linked to short-term mortality. Interestingly, a considerable number of patients were admitted to the ICU from the ED without eliciting an emergency team call. These patients mainly suffered from disability or breathing problems.

The most common circulatory related diagnoses in our emergency team call study population were haemorrhage (11/109), myocardial infarction (9/109) and non-respiratory sepsis (6/109). Severe upper gastrointestinal and post-operative bleeding accounted for half of the haemorrhagic patients (6/11). Prehospital patients diagnosed with STEMI were referred directly to an invasive cardiac centre for PCI according to protocol. Nevertheless, almost half of our MI patients had STEMI or missed STEMI (4/9). Furthermore, three patients sustained cardiac arrest immediately after admission to the ED. All these circulatory problems are associated with a poor prognosis, which may explain the high proportion of circulatory related deaths.

Patients who died within 30 days among admissions eliciting an emergency team call were on average 10 years older than those who survived, and approximately half of the patients who died were deemed ineligible for ICU admission upon initial assessment in the ED. This may indicate a high degree of comorbidity, and some deaths among these patients were presumably expected deaths. Accordingly, ICU LOS was brief, and ICU admittance was not significantly associated with 30-day mortality.

More than half of the emergency team call patients (53.2 %) were admitted to the ICU. Whether more patients should have been admitted to the ICU is a question of resources and ethics. On one hand, older patients with a possible capacity for recuperation of physical and mental health should be offered the maximal level of care possible. On the other hand, patients with various chronic diseases and a poor chance of recovery, may not wish risking to end their lives in an ICU setting. This theme will certainly be of great relevance bearing in mind the increasing percentage of elderly in most Western countries [13].

The review of ICU admissions during the study period identified a considerable number of additional patients, who were admitted to the ICU from the ED without emergency team call activation. In contrast to emergency team call patients, these patients mainly suffered from disability and breathing related problems e.g. intoxication/unconsciousness and COPD exacerbation. These patients required immediate intensive care monitoring and/or treatment, without the need of additional diagnostic assessment in the ED. This may indicate that circulatory related problems were more diagnostically challenging and thus more likely to benefit from an immediate multi-specialty approach, and explain the more frequent use of emergency team calls among such patients.

This study elucidated the characteristics of the most critically ill non-trauma ED patients that elicit emergency team calls, which may bring about improved training of ED personnel and priority of ED resources. Severely critically ill patients were rare in our study, although such patients suffered a wide range of diseases. This highlights the need for emergency physicians to be capable of identifying and initiating relevant treatment to a wide variety of critical conditions before handover or transfer of patients to another setting [14]. This is consistent with the existing structure in our ED, where experienced senior ED physicians are first in line when handling critically ill patients.

Future studies should focus on identifying those critically ill patients who would benefit most from ICU admission, without overwhelming the intensive care services with referrals. For the development of better care pathways a solution may be to target the most frequent conditions that lead to time-critical situations, ICU admissions and deaths, as described by this study. Better targeting of admissions may result in a more effective use of intensive care facilities, with reduced length of stay in both ICU and hospital, and with improved survival. It is advisable to be attentive of the desired level of treatment of elderly patients with severe chronic illnesses, before critical situations arise.

There are several limitations to this study. Firstly, this is a retrospective study, and the decision to elicit emergency team calls may have differed among the coordinating physicians. Secondly, the study cannot provide evidence for the benefit of having this type of emergency team calls, because this was a single centre study without a control group. Emergency team calls could have been reasonable in other cases as well, but we did not scrutinize the medical records of all other admitted patients, because that would require scrutiny of over 27.000 accumulated records. Finally, the low number of critically ill patients in such a cohort may likely reduce the probability of detecting relevant prognostic factors.

## Conclusions

In this study we sought to describe adult non-trauma patients eliciting emergency team calls in a secondary hospital ED. The majority of the patients presented with circulatory, disability and breathing problems, respectively. Half of these patients were admitted to the ICU, although a high proportion of patients (18.3 %) was deemed ineligible for ICU admission. Thirty-day mortality was considerable (31.2 %), and increasing age and circulatory related illnesses were associated with short-term mortality.

## Additional file

Additional file 1:
**Table S3.** Specification of circulatory related diagnoses. (DOC 78 kb)
