# Supplementary material for: Emergency team calls for critically ill non-trauma patients in the emergency department: an observational study
Source: Scand J Trauma Resusc Emerg Med. 2015 Oct 6;23:76. doi: 10.1186/s13049-015-0159-2 (PMC4594893; doi:10.1186/s13049-015-0159-2)
Supplement: Additional file 1: — Table S3. Specification of circulatory related diagnoses. (DOC 78 kb) [file 13049_2015_159_MOESM1_ESM.doc]

**Supplementary material**

**Table 3. Specification of circulatory related diagnoses.**

| Category |  | Diagnosis |  | n |  | % |
| --- | --- | --- | --- | --- | --- | --- |
| Circulation |  |  |  | 41 |  | 37.6 |
|  |  | Haemorrhage |  | 11 |  | 10.1 |
|  |  |  | Post-operative | 3 |  | 2.8 |
|  |  |  | Upper GI | 3 |  | 2.8 |
|  |  |  | Lower GI | 2 |  | 1.8 |
|  |  |  | Ruptured AAA | 1 |  | 0.9 |
|  |  |  | Vaginal bleeding in pregnancy | 1 |  | 0.9 |
|  |  |  | Medical bleeding, anticoagulant therapy | 1 |  | 0.9 |
|  |  | MI |  | 9 |  | 8.3 |
|  |  |  | STEMI | 3 |  | 2.8 |
|  |  |  | Non-STEMI | 4 |  | 3.7 |
|  |  |  | Missed STEMI | 1 |  | 0.9 |
|  |  |  | Type 2 infarction | 1 |  | 0.9 |
|  |  | Sepsis (non-respiratory) | | 6 |  | 5.5 |
|  |  | Arrhythmia |  | 4 |  | 3.7 |
|  |  |  | Ventricular tachycardia | 2 |  | 1.8 |
|  |  |  | Third-degree atrioventricular block | 2 |  | 1.8 |
|  |  | Acute kidney failure |  | 3 |  | 2.8 |
|  |  | Cardiac arrest |  | 3 |  | 2.8 |
|  |  |  | Severe sepsis | 1 |  | 0.9 |
|  |  |  | Pulmonary embolism | 1 |  | 0.9 |
|  |  |  | Unknown | 1 |  | 0.9 |
|  |  | Dehydration |  | 2 |  | 1.8 |
|  |  | Ketoacidosis |  | 2 |  | 1.8 |
|  |  | Hypotension |  | 1 |  | 0.9 |
|  |  |  |  |  |  |  |

GI = gastrointestinal, AAA = Abdominal aortic aneurysm, MI = myocardial infarction, STEMI = ST segment elevation myocardial infarction.
